# Multi-Class Pharmaceutical Profiling Along an Urbanization Gradient in a Tropical Megacity River: Evidence for Cumulative Loading and Limited Attenuation

**DOI:** 10.1007/s00128-026-04271-6

**Published:** 2026-06-16

**Authors:** Wulan Koagouw, Riyana Subandi, Rina Adriany, Richard J. Hazell

**Affiliations:** 1 Research Center for Biota Systems, National Research and Innovation Agency - Republic of Indonesia, KST Soekarno, Jl. Raya Jakarta-Bogor Km.46, Cibinong, Indonesia; 2 Directorate of Research and Innovation Infrastructure, National Research and Innovation Agency - Republic of Indonesia, Jl. M.H. Thamrin No. 8, Jakarta Pusat, 10340 Indonesia; 3 Jakarta Global University, Education Park Jl. Boulevard Grand, Depok, 16412 Indonesia; 4 School of Life Sciences, University of Sussex, Falmer, Brighton, BN1 9RH UK

**Keywords:** Emerging contaminants, Pharmaceutical pollution, Tropical urban rivers, Ciliwung river, Wastewater management

## Abstract

Urban pharmaceutical pollution threatens aquatic ecosystems and human health, yet tropical watersheds remain understudied compared to temperate systems. Here we demonstrate a gradient-based approach to diagnose cumulative pharmaceutical loading in Jakarta’s Ciliwung River. We analyzed 53 compounds across eight sites and detected 13 pharmaceuticals spanning antibiotics, analgesics, cardiovascular agents, and iodinated contrast media (ICMs). Downstream concentrations of the pooled contaminants increased significantly (R^2^ = 0.209, *p* < 0.001), with gabapentin showing the strongest cumulative gradient (R^2^ = 0.895, *p* < 0.001). Eleven compounds displayed monotonic downstream increases, indicating cumulative loading and limited attenuation. Localized spikes for diclofenac and metronidazole suggest point-source inputs from healthcare or residential discharge. The study establishes a baseline for a highly urbanized watershed while illustrating monitoring and source-control priorities relevant to tropical megacities with limited wastewater treatment capacity. It identifies gabapentin as a candidate sentinel indicator for future studies. The findings of this study can aid in development of effective strategies for managing emerging contaminants in the Ciliwung River and other urban rivers facing similar challenges.

**Supplementary Information:**

The online version contains supplementary material available at 10.1007/s00128-026-04271-6.

## Introduction

Pharmaceutical contamination of aquatic ecosystems has emerged as a global environmental challenge, with active pharmaceutical ingredients (APIs) detected in rivers across all continents (Wilkinson et al. 2022). While extensive research has characterized pharmaceutical pollution in temperate urban rivers of Europe, North America, and East Asia, tropical megacity watersheds, particularly in Southeast Asia, remain understudied (Mariano et al. 2023; Bhagat et al. 2024). This knowledge gap is concerning given that tropical rivers exhibit distinct hydrological dynamics and attenuation behaviors compared to temperate systems (Devault et al. 2021), and that many developing tropical cities lack adequate wastewater treatment infrastructure (UN-Water 2024).

Urban river systems receive pharmaceutical inputs via multiple pathways, including treated and untreated wastewater from hospitals, households, and pharmaceutical manufacturing, improper disposal of unused medications, and agricultural runoff (Fairbairn et al. 2016; Ebele et al. 2017). Wastewater treatment plants often fail to remove pharmaceuticals effectively, particularly persistent compounds such as iodinated X-ray contrast media (ICMs), gabapentin, and certain antibiotics (Ebele et al. 2017; Nowak et al. 2020). Once in aquatic environments, pharmaceuticals can persist, bioaccumulate, and exert ecotoxicological effects on aquatic organisms, including endocrine disruption, altered behavior, and contribution to antimicrobial resistance (Fabbri et al. 2016; Guan et al. 2017).

Recent global assessments have identified sub-Saharan Africa, South Asia, and Southeast Asia as regions with the highest pharmaceutical pollution burdens (Wilkinson et al. 2022; Ogunbanwo et al. 2022). Southeast Asian nations face particular challenges: rapid urbanization, poor wastewater treatment infrastructure, high population densities, and tropical conditions that influence contaminant fate, including enhanced biodegradation of some compounds (Anh et al. 2021; Devault et al. 2021). Elevated temperatures in tropical systems may also enhance the biological effects of pharmaceuticals in aquatic organisms; studies on blue mussels (*Mytilus edulis*) have demonstrated synergistic effects of metformin and increased temperature on cellular stress responses and apoptosis induction (Koagouw and Ciocan 2018; Koagouw et al. 2021a), providing direct evidence that temperature modulates pharmaceutical impacts in aquatic invertebrates.

Indonesia, the world’s fourth most populous nation, exemplifies these challenges. Jakarta, with > 30 million inhabitants in the greater metropolitan area, generates vast amounts of wastewater, yet centralized treatment serves less than 5% of the population (Setiawati et al. 2013). The Ciliwung River traverses this megacity, receiving inputs from residential areas, hospitals, commercial zones, and industrial facilities before discharging into Jakarta Bay. Previous studies have documented significant pharmaceutical pollution in Jakarta waters, including detection of metformin at elevated concentrations (Koagouw et al. 2024), and detection of paracetamol at high concentrations in Jakarta Bay (Koagouw et al. 2021b), yet comprehensive multi-class profiling within the river corridor has been lacking, limiting the ability to evaluate cumulative loading patterns. The present study applies a multi-class (i.e., pharmaceuticals spanning multiple therapeutic categories, including antibiotics, analgesics, cardiovascular agents, and iodinated contrast media) target list across an upstream-to-downstream corridor to quantify detection, gradients, and compound-specific spatial signatures, while providing the first comprehensive assessment of these compounds in Indonesia’s most urbanized watershed.

This study’s findings have implications for wastewater management policy, pharmaceutical surveillance strategies, and understanding contaminant transport in tropical urban rivers.

## Methods and Materials

### Study Area and Site Selection

The Ciliwung River originates in Bogor Regency and flows approximately 119 km northward through Bogor, Depok, and Jakarta, discharging into Jakarta Bay (PEMSEA 2025). The watershed covers 337 km^2^ and spans a steep urbanization gradient from forested mountainous upper reaches to densely populated lowlands (Runtunuwu et al. 2010). Population density in Jakarta averages 16–17,000 people/km^2^ overall, with Central Jakarta exceeding 21,000 people/km^2^ (BPS Statistics Indonesia 2025). The river is a major water source for Jakarta and a significant pollution conduit.

**Fig. 1:**
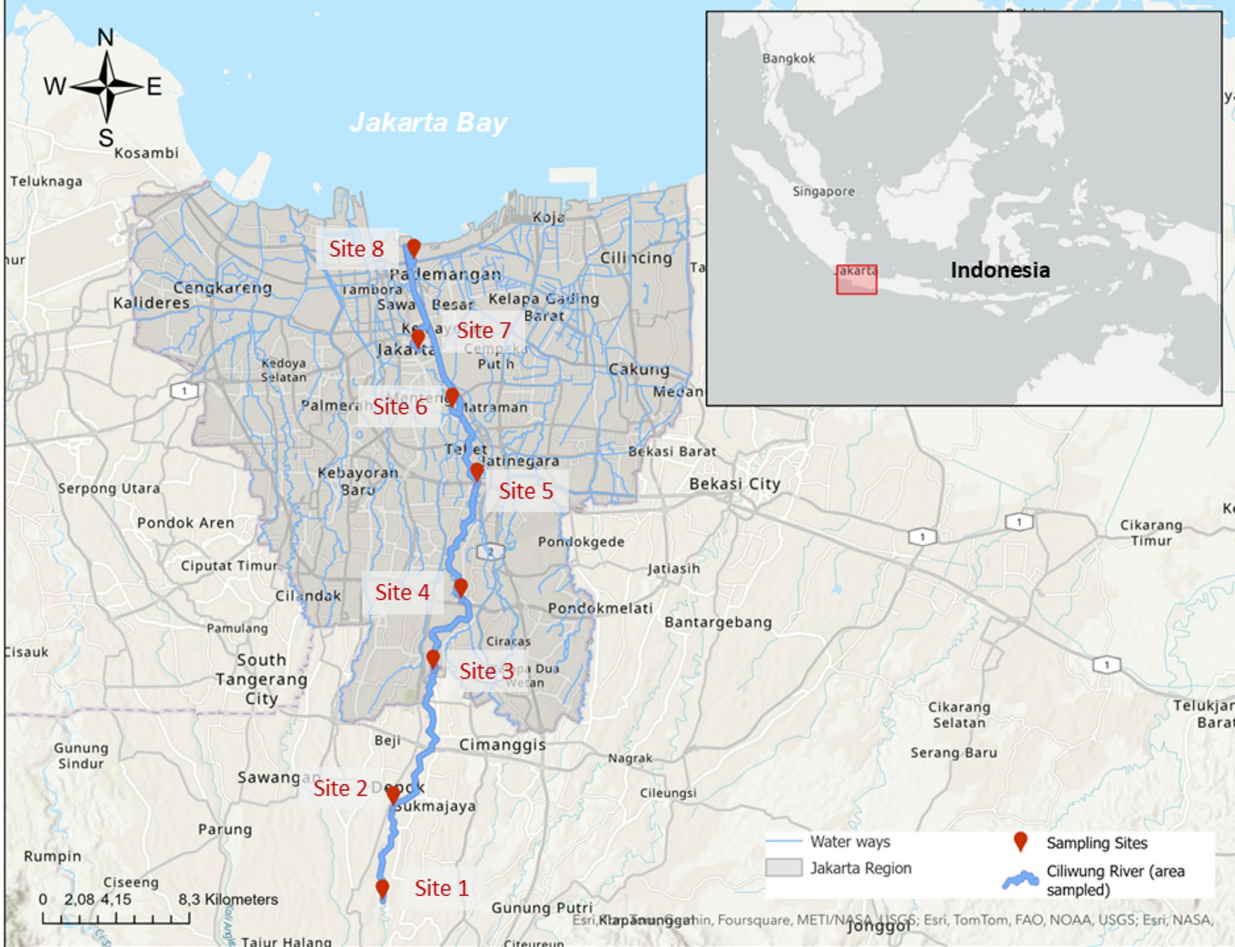
Map of sampling locations along the Ciliwung River. Map of Indonesia (inset) shows location of Jakarta and study area (red box). Maps created in ArcGIS Pro 3.5.2

Eight sampling sites were selected to capture spatial variation in pharmaceutical inputs along an ordered upstream-to-downstream gradient (Fig. 1).

### Sample Collection and Preservation

At each site, composite samples were collected by taking 4–5 sub-samples at 3-meter intervals across the river width over a 5-minute period using a precleaned 2-liter bucket. Sub-samples were pooled, homogenized, and transferred to precleaned 40-mL glass vials (methanol-rinsed, air-dried). Two independent composite samples were collected using the same sub-sampling procedure to assess within-site variability. Reported concentrations represent the mean of the two duplicates. Where elevated variability between duplicate pairs is observed (visible in supplementary Fig. S1), this is attributed to genuine spatial heterogeneity within the river cross-section at those sites. Samples were immediately placed on ice (≤ 4 °C) and transported to the laboratory within 4 h. Sampling was conducted during daylight hours (approximately 07:00–14:00) without stratification for diurnal variation. All samples were collected on the same day, during the transitional period between dry and wet seasons. Water temperature at the time of sampling was 25–29 °C; weather conditions were clear (sunny) to overcast (cloudy). River discharge data were not measured concurrently; this precludes formal mass-flux calculation, as noted in the Statistical Note.

### Analytical Methods

Pharmaceuticals were quantified using liquid chromatography-tandem mass spectrometry (LC-MS/MS: Xevo TQ-XS, Waters, USA; Acquity UPLC system). Samples were filtered (0.7 μm glass fiber filters, Whatman GF/F) and spiked with isotopically labeled internal standards (sulfamethoxazole-^13^C_6_, buprenorphine-D_4_, carbamazepine-D_10_; Cambridge Isotope Laboratories, Cerilliant) to correct for matrix effects. All standards, method blanks, and matrix spikes were processed through GF/F filters in the same manner as field samples, ensuring consistent treatment across all sample types. Direct injection (15 µL) was used following US EPA Method 1694 (US-EPA 2007), eliminating solid-phase extraction to reduce sample loss.

Chromatographic separation used a BEH C18 column (100 × 2.1 mm, 1.7 μm; Waters) with gradient elution (0.4 mL/min, 40 °C). Mobile phases: (A) 0.1% formic acid in water, (B) acetonitrile for ESI+ mode; (A) 5 mM ammonium acetate in water, (B) acetonitrile for ESI− mode. MS/MS operated in Multiple Reaction Monitoring (MRM) mode with optimized transitions for each analyte. Data were processed using MassLynx 4.2 with TargetLynx (Waters).

Quantification employed external calibration (7-point curves, certified reference materials from Cerilliant/MilliporeSigma) with internal standard correction. Reporting limits ranged from 10 to 30 ng/L. Quality assurance included: method blanks (*n* = 3), reagent blanks, matrix spikes, and duplicate analyses. Recoveries ranged from 70 to 130% for target analytes.

### Data Analysis

Statistical analyses were conducted in R version 4.1.2 (R Core Team 2021). Linear regression assessed relationships between site number (proxy for distance along river course, urbanization gradient) and pharmaceutical concentrations. Site number (1–8) served as the independent variable; dependent variables were pharmaceutical compound concentrations. For compounds with below-detection-limit values, we used zero substitution for graphical visualization; regression analyses included only samples with detected concentrations to avoid biasing slope estimates.

Model fit was evaluated using *R²* and *F*-statistics; significance threshold α = 0.05. Normality assumptions were assessed via Q-Q plots and Shapiro-Wilk tests (not shown); log-transformations were not required as concentration distributions were approximately normal or the large sample size (*n* = 208 observations for pooled analysis) invoked the central limit theorem.

### Statistical Note

Site number is an ordinal proxy for spatial position and urbanization intensity but does not represent true physical distance or a continuous predictor. The significant linear relationships indicate monotonic trends but do not imply uniform per-kilometer loading rates. Throughout this manuscript, “cumulative loading” is used as a conceptual descriptor of progressive downstream accumulation of pharmaceutical concentrations; it does not represent formal mass-flux load calculations, which would require concurrent discharge measurements not obtained in this study. Future studies incorporating precise geospatial data would enable mechanistic modeling of pharmaceutical transport. The baseline established here provides a foundation for targeted follow-up studies addressing temporal variability and mechanistic processes.

## Results

### Detection Frequency and Concentration Ranges

Of the 53 pharmaceuticals investigated (Table S1), 13 compounds were detected above method detection limits (Table 1, Fig. S1). Detection frequencies varied markedly among therapeutic classes: five antibiotics (chloramphenicol, lincomycin, metronidazole, sulfamethoxazole, trimethoprim), three analgesics (diclofenac, paracetamol, tramadol), valsartan, gabapentin, and three ICMs (iohexol, iopamidol, iopromide).

**Table 1:** Mean and cumulative concentrations of pharmaceuticals detected at each site

Pharmaceutical	Detection Limit (ng/L)	Concentration (ng/L)*								Mean Conc. (ng/L)**	Cumulative Conc. (ng/L)
		Site 1	Site 2	Site 3	Site 4	Site 5	Site 6	Site 7	Site 8		
Chloramphenicol	10	< 10	< 10	< 10	< 10	17	17	22.5	18.5	18.8	75
Diclofenac	10	< 10	16.5	< 10	< 10	13	15.5	22.5	17.5	17	85
Gabapentin	10	18.5	25.5	33	43	47.5	57.5	97.5	92.5	51.9	415
Iohexol	30	< 30	< 30	< 30	< 30	30	37.5	151.5	102	80.3	321
Iopamidol	30	< 30	< 30	< 30	< 30	< 30	< 30	48.5	99	73.8	147.5
Iopromide	30	< 30	< 30	< 30	< 30	< 30	< 30	236	120.5	178.3	356.5
Lincomycin	10	< 10	< 10	< 10	< 10	< 10	< 10	12	24	18	36
Metronidazole	10	< 10	53.5	< 10	< 10	< 10	< 10	13.5	17	28	84
Paracetamol	10	23	15	11	< 10	32.5	17.5	69.5	< 10	28.1	168.5
Sulfamethoxazole	10	14.5	14	12	15	20	34	48.5	75.5	29.2	233.5
Tramadol	10	18	26.5	25	27.5	35.5	35	49.5	58	34.4	275
Trimethoprim	10	< 10	< 10	< 10	< 10	< 10	10	13	12	11.7	35
Valsartan	10	< 10	< 10	< 10	< 10	< 10	10.5	15.5	30.5	18.8	56.5
Site Total (ng/L)	-	74	151	81	85.5	195.5	234.5	800	667	-	-

* Values represent the mean of two independently collected duplicate samples per site.

** Mean concentration across detection sites, calculated as the arithmetic mean of site-level concentrations at sites where the compound was detected.

Detected concentrations ranged from 10 ng/L (at or near the reporting limit) to 236 ng/L (iopromide, Site 7), spanning approximately one to two orders of magnitude across individual compounds and sites. ICMs exhibited the highest individual site concentrations: iopromide (mean 178.3 ng/L calculated across the two sites where detection occurred, maximum 236 ng/L at Site 7), iohexol (mean 80.3 ng/L across four detection sites, maximum 151.5 ng/L at Site 7), and iopamidol (mean 73.8 ng/L across two detection sites, maximum 99 ng/L at Site 8). Among non-ICM pharmaceuticals, gabapentin showed the highest mean concentration (51.9 ng/L) and was detected at all eight sites. Sulfamethoxazole (mean 29.2 ng/L) and tramadol (mean 34.4 ng/L) were likewise detected at all sites. In contrast, certain compounds exhibited sporadic detection: chloramphenicol, lincomycin, trimethoprim, and valsartan were detected at ≤ 4 sites, suggesting more localized sources or lower environmental persistence.

### Spatial Distribution and Downstream Gradients

**Fig. 2:**
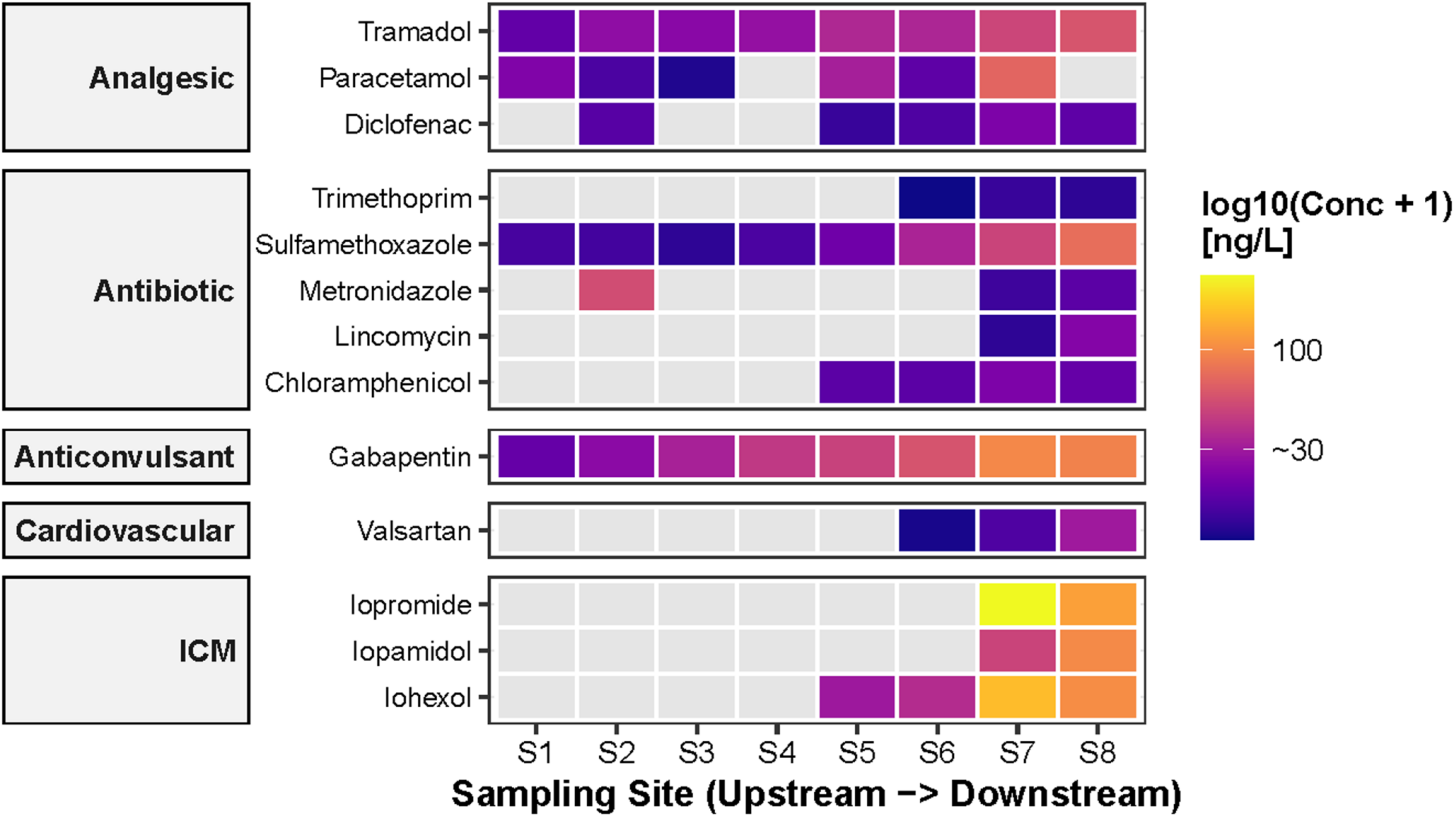
Heatmap showing detection pattern of pharmaceutical across sites (sites × compounds). Color intensity represents concentration, with yellow indicating high and dark blue indicating low concentration

A clear spatial pattern emerged along the river course. Site 1 (upstream) exhibited the lowest pharmaceutical burden (74 ng/L cumulative concentration, 4 compounds), whereas Sites 7 and 8 (central and downstream) showed the highest contamination (800 and 667 ng/L cumulative, 13 and 12 compounds detected respectively; Fig. 2). Linear regression of pooled pharmaceutical concentrations against site number revealed a strong positive relationship (*R*^2^ = 0.209, *F*_1,206_ = 55.8, *p* < 0.001), indicating an increase along the river continuum.

**Fig. 3:**
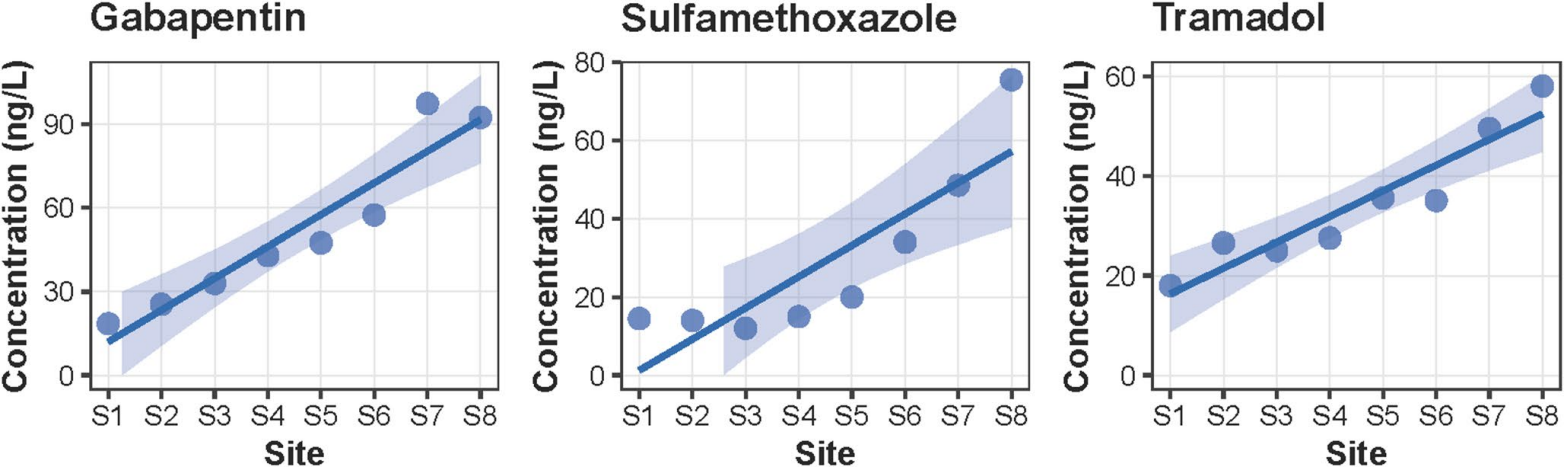
Individual compound regressions for pharmaceuticals showing the strongest downstream increases, with 95% CI bands represented by the blue shaded areas

Compound-specific regressions demonstrated monotonic downstream increases for 11 of 13 detected pharmaceuticals (full regression statistics in Table S2; regressions in Fig. S1). The strongest gradients were observed for gabapentin (*R*^2^ = 0.895, *p* < 0.001), tramadol (*R*^2^ = 0.861, *p* < 0.001), and sulfamethoxazole (*R*^2^ = 0.731, *p* < 0.001) (Fig. 3). ICMs likewise showed strong downstream concentration increases: iohexol (*R*^2^ = 0.622, *p* < 0.001), iopamidol (R² = 0.520, *p* < 0.001), and iopromide (*R*^2^ = 0.409, *p* = 0.005). Only paracetamol and metronidazole failed to exhibit significant linear trends. Metronidazole showed a pronounced concentration spike at Site 2 (53.5 ng/L) relative to all other sites, indicating a localised point source. Paracetamol exhibited an irregular, non-monotonic detection pattern (present at Sites 1–3, 5–7 but below the reporting limit at Sites 4 and 8) which precluded a significant linear trend.

### Cumulative Pharmaceutical Concentrations and Compound Contributions

Across all sites, gabapentin exhibited the highest cumulative concentration (415 ng/L), followed by iopromide (356.5 ng/L), iohexol (321 ng/L), tramadol (275 ng/L), and sulfamethoxazole (233.5 ng/L). This ranking differs from single-site comparisons due to gabapentin’s ubiquitous detection versus ICMs’ more limited spatial occurrence.

### Point-Source Signatures

Diclofenac and metronidazole exhibited localized concentration spikes at Site 2 (16.5 and 53.5 ng/L respectively), contrasting with their low concentrations at other sites. Site 2, located beneath a suspension bridge in Depok, receives runoff from nearby commercial and residential areas. The elevated concentrations of diclofenac (a widely used NSAID) and metronidazole (an antibiotic) suggest potential inputs from healthcare facilities, pharmacies, or concentrated residential wastewater discharge at this location. Such localized signatures are valuable for targeted source-control interventions.

## Discussion

The present study applies systematic pharmaceutical profiling of an urbanization gradient along the Ciliwung River. It reveals widespread contamination with significant downstream accumulation, highlighting key challenges for tropical megacity watersheds with limited wastewater treatment capacity.

The downstream increase in pharmaceutical concentrations indicates cumulative loading along the river course, with limited in-stream attenuation. Unlike many temperate rivers where greater seasonal flow variability can provide dilution (Burns et al. 2018) and where higher wastewater treatment coverage reduces pharmaceutical inputs (Johnson et al. 2015), continuous pharmaceutical discharge in tropical rivers with minimal treatment capacity may exceed the river’s natural attenuation capacity (Setiawati et al. 2013). Jakarta’s wastewater management faces systemic challenges: <5% of the population is connected to centralized treatment, most households rely on often poorly maintained septic tanks, and informal settlements discharge untreated sewage directly into waterways (Setiawati et al. 2013). Globally, 42% of household wastewater received inadequate or no treatment in 2022, with 113 billion m³ released to the environment (UN-Water 2024). In Jakarta, this fraction likely exceeds 90% (Roberts et al. 2019), resulting in continuous pharmaceutical loading to the Ciliwung River. The scale of the issue necessitates both upgrading and expanding centralized treatment while implementing decentralized solutions such as improved septic systems, complemented by pharmaceutical take-back programs and public education on proper disposal.

The pharmaceutical contamination profile also reflects multiple source categories with distinct spatial patterns. The concentration of ICMs at Sites 5–8, coupled with the highest overall pharmaceutical burdens at Sites 7–8 (central Jakarta with numerous hospitals), implicates healthcare wastewater as a major source. ICMs are extensively used in medical imaging and are highly persistent in aquatic environments due to their complex chemical structures and resistance to biological degradation, with conventional wastewater treatment plants achieving < 20% removal efficiency (Nowak et al. 2020; Yan et al. 2024). Studies in the Philippines detected metformin, iopamidol, sulfamethoxazole, ciprofloxacin, and azithromycin in untreated hospital wastewater but not in other water bodies, confirming hospitals as point sources (Mariano et al. 2023). Gabapentin, tramadol, and paracetamol are commonly used outpatient medications, with their widespread detection reflecting diffuse residential inputs along the river course. Spikes in diclofenac and metronidazole at Site 2 (Depok) suggest a concentrated source, potentially a pharmacy, clinic, or compromised septic system serving a residential cluster. Such localized signatures necessitate targeted intervention, such as pharmaceutical-specific wastewater pretreatment or controlled discharge protocols to reduce iodinated contrast media and antibiotic loads.

Gabapentin emerged as a compound of particular interest. It was detected at all eight sites and had the highest cumulative concentration of all pharmaceuticals detected. Gabapentin is ubiquitously detected in several European aquatic environments and has been shown to produce stable transformation products under various redox conditions (Henning et al. 2018). Its increasing prescription rates across many countries globally identify it as an emerging pharmaceutical contaminant of broad concern (Chan et al. 2023). Therefore we propose that gabapentin warrants consideration as a sentinel indicator for pharmaceutical pollution in tropical urban rivers, subject to further validation. It satisfies most of the criteria – widespread environmental detection, persistence, correlation with overall contamination patterns, measurability with standard analytical methods, and an ecotoxicological threshold context. A recent study demonstrates that gabapentin exposure at environmentally relevant concentrations (1–100 µg/L) causes reproductive toxicity, endocrine disruption, and developmental effects in zebrafish (He et al. 2025), providing a foundation for risk-based monitoring thresholds.

Among the pharmaceuticals detected in the present study, antibiotics (e.g., sulfamethoxazole, trimethoprim) and NSAIDs (diclofenac) have documented effects on fish in laboratory studies, including reproductive impairment, altered embryo development, renal failure, and behavioral changes (Brausch et al. 2012; Bielen et al. 2017; Huang et al. 2025). Gabapentin and tramadol have demonstrated ecotoxicological effects in controlled laboratory studies at µg/L concentrations (He et al. 2025; Santos et al. 2021). The concentrations detected in the present study (gabapentin up to 97.5 ng/L and tramadol up to 58 ng/L) are approximately 10- to 17-fold below the lowest published effect concentrations, suggesting that individual-compound risk at current detected levels may be limited. However, the simultaneous presence of multiple pharmaceuticals raises the possibility of additive or synergistic mixture effects, which remain uncharacterised for this compound assemblage and represent an important research priority.

Given the Ciliwung River’s discharge into Jakarta Bay, pharmaceutical contaminants represent an additional stressor to already-impacted coastal ecosystems, with potential cascading effects on fisheries and marine biodiversity. The ecological consequences for Jakarta Bay’s fisheries and mangrove ecosystems remain understudied and warrant further investigation. Additionally, the 53 compounds investigated in the present study are not exhaustive. For example, the potential occurrence in the Ciliwung River corridor of metformin, not included here but detected at concentrations up to 414 ng/L in the adjacent Angke River (Koagouw et al. 2024), represents a possible future avenue for comparative research.

This baseline assessment provides essential data for developing evidence-based pharmaceutical pollution policies in Indonesia and serves as a model for similar studies in understudied tropical megacity watersheds. More generally, the combination of multi-class target screening, gradient-based site selection, and monotonic trend evaluation provides a transferable template for diagnosing cumulative pharmaceutical loading in other urban river systems. Future research priorities include: (1) temporal variability assessment (wet vs. dry season); (2) ecotoxicological risk assessment for detected pharmaceuticals in tropical freshwater and marine species; (3) source apportionment using compound ratios and spatial signatures; and (4) evaluation of low-cost treatment technologies feasible for tropical settings.

## Electronic Supplementary Material

Below is the link to the electronic supplementary material.

Supplementary Material

## Data Availability

All data supporting the findings of this study are available in the main text, figures, tables, and Supplementary Information, or upon request.
