# Supplementary material for: Multi-Class Pharmaceutical Profiling Along an Urbanization Gradient in a Tropical Megacity River: Evidence for Cumulative Loading and Limited Attenuation
Source: Bull Environ Contam Toxicol. 2026 Jun 16;117(1):1. doi: 10.1007/s00128-026-04271-6 (PMC13272248; doi:10.1007/s00128-026-04271-6)
Supplement: Supplementary file 1 — Supplementary Material [file 128_2026_4271_MOESM1_ESM.docx]

**Supplementary Information: Multi-class pharmaceutical profiling along an urbanization gradient in a tropical megacity river: evidence for cumulative loading and limited attenuation – Koagouw et al.**


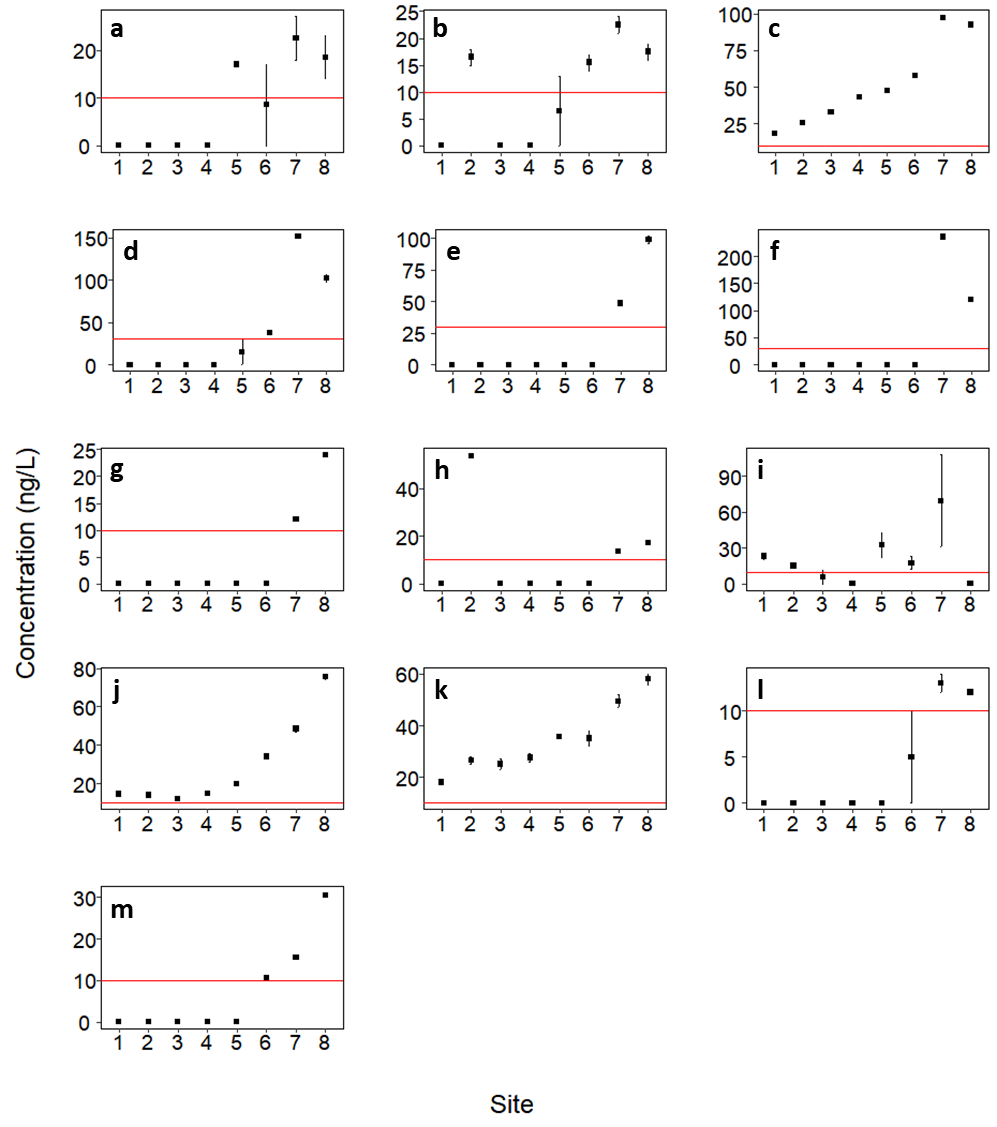


**Supplementary Figure S1.** Plots showing per-site concentrations of each individual pharmaceutical, represented here by letter (a: Chloramphenicol, b: Diclofenac, c: Gabapentin, d: Iohexol, e: Iopamidol, f: Iopromide, g: Lincomycin, h: Metronidazole, i: Paracetamol, j: Sulfamethoxazole, k: Tramadol, l: Trimethoprim, m: Valsartan). Black squares represent mean values, while whiskers represent upper and lower concentrations recorded, if different. Zeroes represent values below the limit of detection, displayed here as red horizontal lines.

**Supplementary Table S1.** Retention Times, MRM Transitions, and Quantification Limits for Target Pharmaceuticals in River Water Using LC-MS/MS

| **Analyte** | **RT (min)** | **Parent → Daughter (m/z)** | **ESI Mode** | **LOR (µg/L)** |
| --- | --- | --- | --- | --- |
| Sulfamethoxazole | 2.51 | 254 → 156 | ESI− | 0.01 |
| Buprenorphine | 1.80 | 468 → 83 | ESI+ | 0.01 |
| Iopromide | 1.50 | 791 → 127 | ESI− | 0.03 |
| Iomeprol | 1.50 | 777 → 127 | ESI− | 0.03 |
| Hydrochlorothiazide | 2.30 | 296 → 204 | ESI− | 0.01 |
| Enalapril | 1.23 | 377 → 234 | ESI+ | 0.01 |
| Cyclobenzaprine | 4.10 | 276 → 231 | ESI+ | 0.01 |
| Capecitabine | 11.50 | 360 → 128 | ESI+ | 0.01 |
| Butorphanol | 2.00 | 328 → 98 | ESI+ | 0.01 |
| Azathioprine | 5.50 | 278 → 162 | ESI+ | 0.01 |
| Anastrozole | 6.50 | 294 → 225 | ESI+ | 0.01 |
| Zolpidem | 1.76 | 308 → 235 | ESI+ | 0.01 |
| Metronidazole | 2.95 | 172 → 128 | ESI+ | 0.01 |
| Tramadol | 3.50 | 264 → 58 | ESI+ | 0.01 |
| Oxazepam | 3.00 | 287 → 241 | ESI+ | 0.01 |
| Salbutamol | 3.60 | 240 → 222 | ESI+ | 0.01 |
| Piroxicam | 7.40 | 332 → 95 | ESI− | 0.01 |
| Mycophenolate Mofetil | 11.20 | 434 → 191 | ESI+ | 0.01 |
| Metoprolol | 6.30 | 268 → 191 | ESI+ | 0.01 |
| Ketoprofen | 6.60 | 254 → 206 | ESI− | 0.01 |
| Iopamidol | 1.50 | 777 → 127 | ESI− | 0.03 |
| Iohexol | 1.60 | 791 → 127 | ESI− | 0.03 |
| Furosemide | 4.90 | 330 → 285 | ESI− | 0.01 |
| Diclofenac | 7.50 | 296 → 214 | ESI− | 0.01 |
| Carbamazepine | 3.90 | 237 → 194 | ESI+ | 0.01 |
| Valsartan | 6.80 | 436 → 180 | ESI− | 0.01 |
| Naproxen | 6.70 | 229 → 185 | ESI− | 0.01 |
| Thebain | 4.40 | 310 → 265 | ESI+ | 0.01 |
| Paracetamol | 3.41 | 152 → 110 | ESI+ | 0.01 |
| Flutamide | 7.50 | 276 → 229 | ESI+ | 0.01 |
| Ciprofloxacin | 1.79 | 332 → 231 | ESI+ | 0.03 |
| Clofibric Acid | 6.00 | 213 → 127 | ESI− | 0.01 |
| Citalopram | 7.30 | 325 → 109 | ESI+ | 0.01 |
| Sertraline | 2.19 | 306 → 159 | ESI+ | 0.01 |
| Indomethacin | 8.20 | 358 → 139 | ESI− | 0.01 |
| Paclitaxel | 11.40 | 854 → 286 | ESI+ | 0.01 |
| Fluoxetine | 2.40 | 310 → 148 | ESI+ | 0.01 |
| Diazepam | 7.90 | 285 → 193 | ESI+ | 0.01 |
| Loperamide | 8.50 | 477 → 259 | ESI+ | 0.01 |
| Ifosfamide | 5.10 | 261 → 90 | ESI+ | 0.01 |
| Gabapentin | 2.30 | 172 → 137 | ESI+ | 0.01 |
| Chloramphenicol | 6.20 | 321 → 152 | ESI+ | 0.01 |
| Lincomycin | 4.80 | 407 → 126 | ESI+ | 0.01 |
| Gemfibrozil | 7.10 | 249 → 121 | ESI− | 0.02 |
| Sulfamethazine | 6.40 | 279 → 186 | ESI+ | 0.01 |
| Cyclophosphamide | 4.20 | 261 → 143 | ESI+ | 0.01 |
| Atenolol | 3.70 | 267 → 145 | ESI+ | 0.01 |
| Warfarin | 7.10 | 307 → 117 | ESI− | 0.01 |
| Trimethoprim | 5.90 | 291 → 230 | ESI+ | 0.01 |
| Terbutaline | 4.30 | 226 → 208 | ESI+ | 0.01 |
| Sotalol | 3.90 | 273 → 254 | ESI+ | 0.01 |
| Propranolol | 4.20 | 260 → 117 | ESI+ | 0.01 |
| Bezafibrate | 6.90 | 361 → 146 | ESI− | 0.01 |

**Supplementary Table S2.** Full statistical results for per-site pharmaceutical concentrations.

| **Pharmaceutical** | **Multiple R-squared** | **Adjusted R-squared** | **F-statistic** | **Degrees of Freedom** | **p-value** |
| --- | --- | --- | --- | --- | --- |
| Chloramphenicol | 0.626 | 0.599 | 23.43 | 1, 14 | <0.001 * |
| Diclofenac | 0.388 | 0.344 | 8.87 | 1, 14 | 0.010 * |
| Gabapentin | 0.902 | 0.896 | 129.4 | 1, 14 | <0.001 * |
| Iohexol | 0.647 | 0.622 | 25.65 | 1, 14 | <0.001 * |
| Iopamidol | 0.552 | 0.52 | 17.22 | 1, 14 | 0.001 * |
| Iopromide | 0.449 | 0.409 | 11.39 | 1, 14 | 0.005 * |
| Lincomycin | 0.555 | 0.523 | 17.43 | 1, 14 | 0.001 * |
| Metronidazole | 0.016 | 0.054 | 0.23 | 1, 14 | 0.641 |
| Paracetamol | 0.036 | 0.037 | 0.53 | 1, 14 | 0.480 |
| Sulfamethoxazole | 0.749 | 0.731 | 41.66 | 1, 14 | <0.001 * |
| Tramadol | 0.871 | 0.861 | 94.2 | 1, 14 | <0.001 * |
| Trimethoprim | 0.637 | 0.611 | 24.52 | 1, 14 | <0.001 * |
| Valsartan | 0.702 | 0.68 | 32.91 | 1, 14 | <0.001 * |
